# Optimal utilization of ecological economic resources and low-carbon economic analysis from the perspective of Public Health

**DOI:** 10.3389/fpubh.2023.1152809

**Published:** 2023-03-23

**Authors:** Qi Wang, Yan Feng, Ao Hu

**Affiliations:** ^1^Management School, Hunan City University, Yiyang, China; ^2^Local Governance Research Institute, Central South University, Changsha, China

**Keywords:** Public Health, ecological economy, Low-Carbon Economy, public spaces in settlements, satisfaction evaluation indicators

## Abstract

**Introduction:**

China's urbanization process continues to deepen with social development, but the optimal utilization of ecological, economic resources and Public Health (PH) problems are becoming increasingly severe.

**Methods:**

This paper analyses the optimal use of urban resources based on PH. Here, the public space of urban settlements is selected as the research object. Firstly, the connotation and essence of the ecological economy and Low-Carbon Economy (LCE) are analyzed. Secondly, the characteristics of public space in urban settlements are studied based on PH. The public space satisfaction evaluation model in urban settlements is constructed with five first-level and 12 second-level indicators. Finally, a questionnaire is designed to analyze urban households' outdoor activities and evaluate public space in settlements.

**Results:**

The influencing factors of residents' satisfaction with public space in settlements are obtained through regression analysis. The results show that residents' satisfaction with the public space of the settlement is mainly evaluated from three aspects: the accessibility of public space, the integrity of public space, and the pleasure of public space. The influence coefficients are 0.355, 0.346, and 0.223, respectively, indicating that the influence degree of the three principal factors decreases in turn.

**Conclusion:**

We can optimize the utilization of urban residential public space resources from the aspects of accessibility, integrity and pleasure, so as to promote residents to go to public spaces for outdoor activities and physical exercise, which is more conducive to the public health of residents.

## 1. Introduction

With the continuous progress of Chinese society, urbanization is also deepening. The focus of urban problems has shifted from focusing on static physical space problems to emotional behavioral, social, temporal and spatial issues. This is followed by a contradiction between the growing needs of residents and the physical spatial environment (1). Therefore, it is essential to study the interaction between Public Health (PH) status and specific spaces that affect health activities, which is one of the characteristics of the quality of life of urban residents. Studies in recent years have shown that China's PH problems have gradually shifted to chronic diseases, sub-health, and sudden infectious diseases. Regular outdoor activities can improve PH and reduce disease risk (2).

The issuance of relevant national documents on the construction of ecological civilization puts forward higher requirements for protecting regional ecological security, optimizing the pattern of spatial development, and protecting and expanding ecological space. The concept of the Low-Carbon Economy (LCE) and ecological economy also means that building an ecological civilization, improving the ecological environment, and promoting green development have become the country's strategic needs in the new era. As the world's largest developing country, China is responsible for reducing carbon emissions and developing an ecological economy (3, 4). LCE can promote the protection and recycling of the ecological environment. A green ecological environment can make people's physical and mental health develop slowly in a positive direction, which is more conducive to PH. Therefore, exploring the optimization and utilization of resources suitable for China's ecological and LCE and achieving sustainable economic and social development is of great practical significance.

Based on this, this paper takes the public space of urban settlements as the research object. It analyzes the characteristics of public space in urban settlements from the perspective of PH from the relevant theories of the ecological economy and LCE. Besides, the evaluation model of public space satisfaction in urban settlements is constructed. The research data are obtained through the questionnaire survey. The data are analyzed by Multiple Linear Regression (MLR) to get the influencing factors affecting residents' outdoor activities in public spaces. The research on the evaluation indicator of public space in urban settlements can provide a theoretical basis for the optimal utilization of public space resources under the ecological economy. The structure of this paper can be divided into the following sections. Section 1 is the introduction that mainly introduces the research's background, purpose, and significance. Section 2 is a literature review. The main questions are raised. Section 3 is the research methodology. It introduces the theoretical foundations used here, such as ecological economy, LCE, PH, and urban public space. The satisfaction evaluation model of public space in settlements is constructed based on the characteristics of public space in urban settlements. Section 4 is experimental design and result analysis. The survey results are obtained by studying the data from the questionnaire by analyzing the source of the data and linear regression. It is compared with similar studies to confirm the correctness of the results. Section 5 is the conclusion that summarizes the results and draws conclusions and the implications and prospects for future research.

## 2. Literature review

Scholars have done much research on public spaces in urban settlements. Shojai and Fattahi explored urban design issues at the micro-urban design level. A comparison was made between Shiraz, Iran, and Sapporo, Japan. They used social psychology methods to study the similarities and differences in the arrangement of open spaces in contemporary settlements (5). Abdallah and Mahmoud aimed to evaluate and improve the thermal comfort of open spaces and the thermal perception of residents in the urban residential community of New Asyut City, Egypt. They assessed the thermal conditions of the periphery of urban residential communities using six design scenarios. It was concluded that increasing tree density and semi-shade in open spaces between residential buildings could increase the external environmental benefits of residents of new urban residential communities in hot arid climates (6). Lai et al. comprehensively reviewed current outdoor thermal comfort research, including outdoor thermal comfort benchmarks, data collection methods, and models. A conceptual framework was proposed. Physical, physiological, and psychological factors acted as direct effects. Behavioral, personal, social, and cultural factors and hot history, places, and allies were indirect influences. Then, these direct and indirect factors were decomposed and reviewed, and the interactions between various factors were discussed (7). Liang et al. proposed a new open-space simulation model using cellular automata. It allowed the spatial simulation of open spaces in urban areas to interact with urban dynamics. It was constrained by strategies to control different average sizes of open space in new urban areas. Walkability and population coverage were used to assess the effectiveness of creating new operating systems (8). Wei and Jones explored the emergence of urban agriculture and its changing nature and role in China's urbanization process. They cited a planned settlement in Kunming, Yunnan Province, as an example. The physical and spatial manifestations of urban farming practices and stakeholder motivations and attitudes were identified (9). For the outdoor thermal comfort and adaptive behavior in the edge season, Leng et al. selected three representative residential public open spaces in Harbin, a typical winter city, for empirical research. Meteorological measurements and basic questionnaires were conducted. Observations were made to explore outdoor thermal comfort and adaptive behavior (10).

The above studies analyze the public space of residential areas from the perspectives of residential area layout, thermal comfort of residential, public space, open space simulation, and outdoor thermal comfort. However, few studies are based on public space in settlements under PH and ecological economy. Therefore, this paper analyzes the satisfaction evaluation model of public space in urban settlements based on public space from ecological economy and LCE.

## 3. Research methodology

### 3.1. Analysis of ecological economy and LCE

Ecological economics is the study of the relationship between ecosystems and economic systems. It plays a vital role in many important issues facing human development today. The core of the study of ecological economics is the issue of sustainable development. The underlying idea is that the earth's economy is a material economy with a closed ecological loop (11). Based on the environmental economy, scholars also proposed the green economy, circular economy, and LCE. The green economy advocates the efficient and civilized use of natural resources based on social and ecological conditions. It is an “affordable economy” with an improved environment and promoted quality of life. Circular economy believes that economy and ecology are a closed-loop and recyclable system. An LCE is about saving energy while reducing greenhouse gas emissions, maintaining economic and social stability, and achieving sustainable development. LCE is developed based on the ecological economy, its ultimate goal (12). A comparison of the four economies is shown in Table 1 (13).

**Table 1:** Comparison of ecological economy, green economy, circular economy, and LCE.

**Conception**	**Ecological economy**	**Green economy**	**Circular economy**	**LCE**
Time of presentation	Late 60s of the 20th century	In 1989	In 1990	In 2006
Theoretical basis	Ecology and economics	Environmental economics, sociology, ecology	Ecological economics	Energy economics
Purpose	Develop a sustainable economy and reduce environmental pollution	The healthy and harmonious development of human society	Build a resource recycling and efficient utilization system	Improve energy efficiency and reduce greenhouse gas emissions

Regardless of the main problems these economies solve, their essence is to promote economic development, ecological protection, and social progress. The ultimate goal is to achieve sustainable development of human society (14).

### 3.2. Public spaces in urban settlements under PH

Health was initially considered and mechanically to be a state of “absence of disease and disability.” With the development of medicine, the connotation of health is also gradually developing. The World Health Organization defines “health” as not simply the absence of any disease or infirmity of an individual but a state in which they adapt well to the society of this era at physical, mental, and social levels (15). PH refers to the overall health of the population. The collective health requires the coordination of defense mechanisms with the whole society. PH includes three aspects: physical, mental, and social health. Among them, physical health refers to the good physical fitness of residents, and there are no diseases. Mental health refers to residents' emotional and mental stability without negative emotions such as anxiety and depression. Social health is the harmonious and good interpersonal relationship generated by the interaction between the individual resident and the physical space environment and social environment (16).

**Figure 1:**
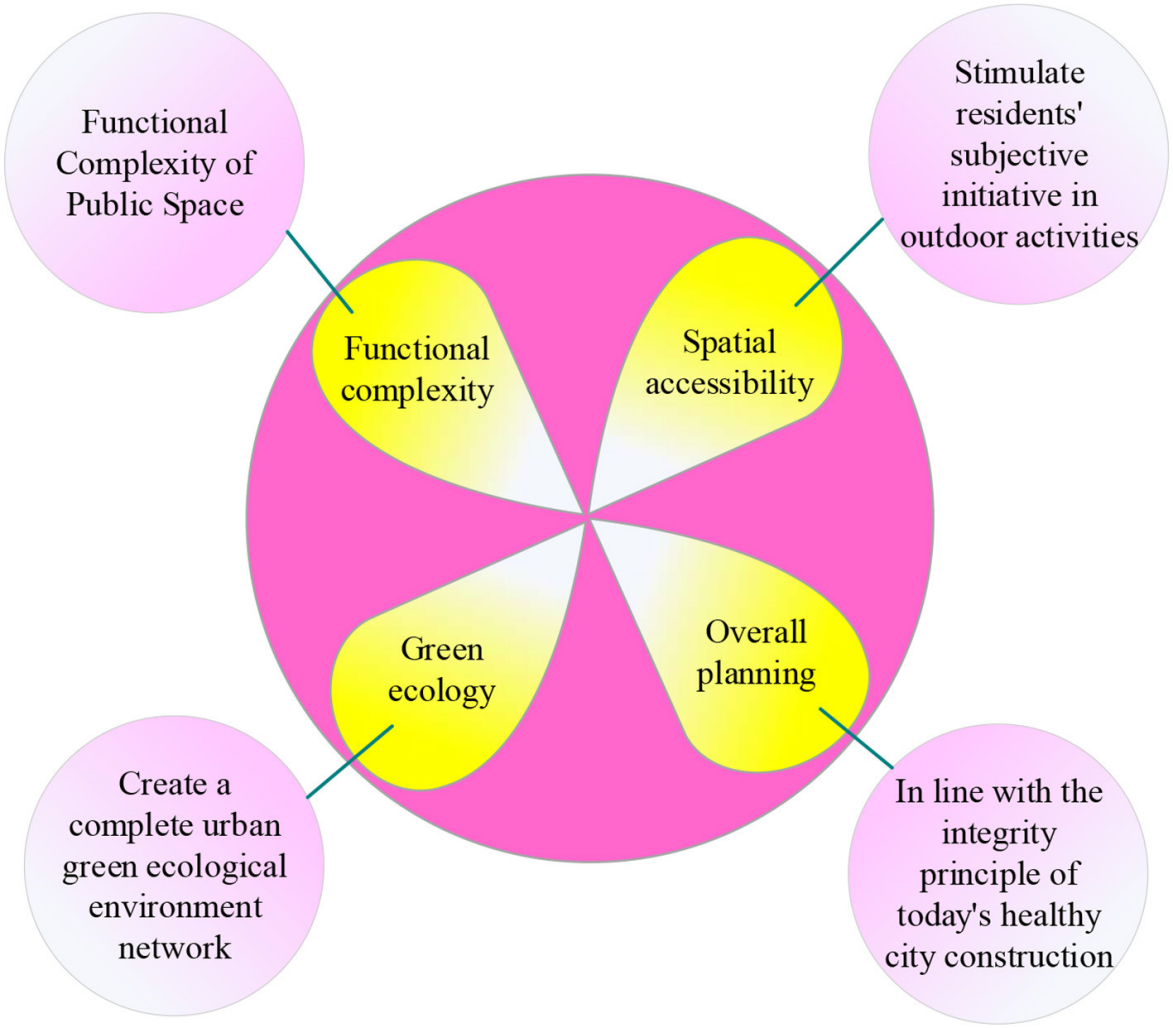
Characteristics of public space in urban settlements.

Cities are where residents live and work, and settlements are relevant to everyone. The public space of the residential regions is an important material function space that carries residents' daily activities (17). The public space of urban settlements is the spatial carrier of residents' activities. Its core value is publicity. It includes physical space and a multi-level system consisting of functional facilities, green spaces, and traffic spaces. The public space of urban settlements is the generator of residents' daily life. It functions as a public service and public activity in settlements and provides residents with ubiquitous leisure spaces (18, 19). Public space in urban settlements needs to be well utilized as a spatial resource in the ecological and LCE. However, the rapid development of society has led to urban environmental pollution. The quality of the space deteriorates. The sedentary way of working of modern people leads to reduced physical activity. These create an imbalance between supply and demand (20). As a medium, the public space of urban settlements has the extroversion of communicating urban space and can carry the function of residents' daily life. It has the introversion of aggregating residents and neighbors. The main characteristics of public spaces in urban settlements are shown in Figure 1 (21, 22).

Figure 1 shows that public space in urban settlements has four characteristics: functional complexity, spatial accessibility, green ecology, and planning coordination (23, 24). First, residents' use of public spaces in settlements is often not limited to a single function. For example, parents will chat with neighbors while babysitting in the children's play space. Seniors will square dance on unused basketball courts, which requires the functional complexity of public spaces. Second, residents consider the accessibility of the space to decide whether to go to the space to participate in the activities they want to join. A high level of accessibility stimulates residents' initiative to engage in outdoor activities actively. Then, as the functional group of the smallest unit, the public space of urban settlements is the public space closest to the lives of residents. It is an integral part of the urban green ecological environment network to improve the green network and create a complete system. It has green ecology. Finally, promoting public space construction in urban settlements under the guidance of PH should form a systematic public space system which meets the overall principle of today's healthy city construction.

### 3.3. Satisfaction evaluation model of public space in settlements

**Figure 2:**
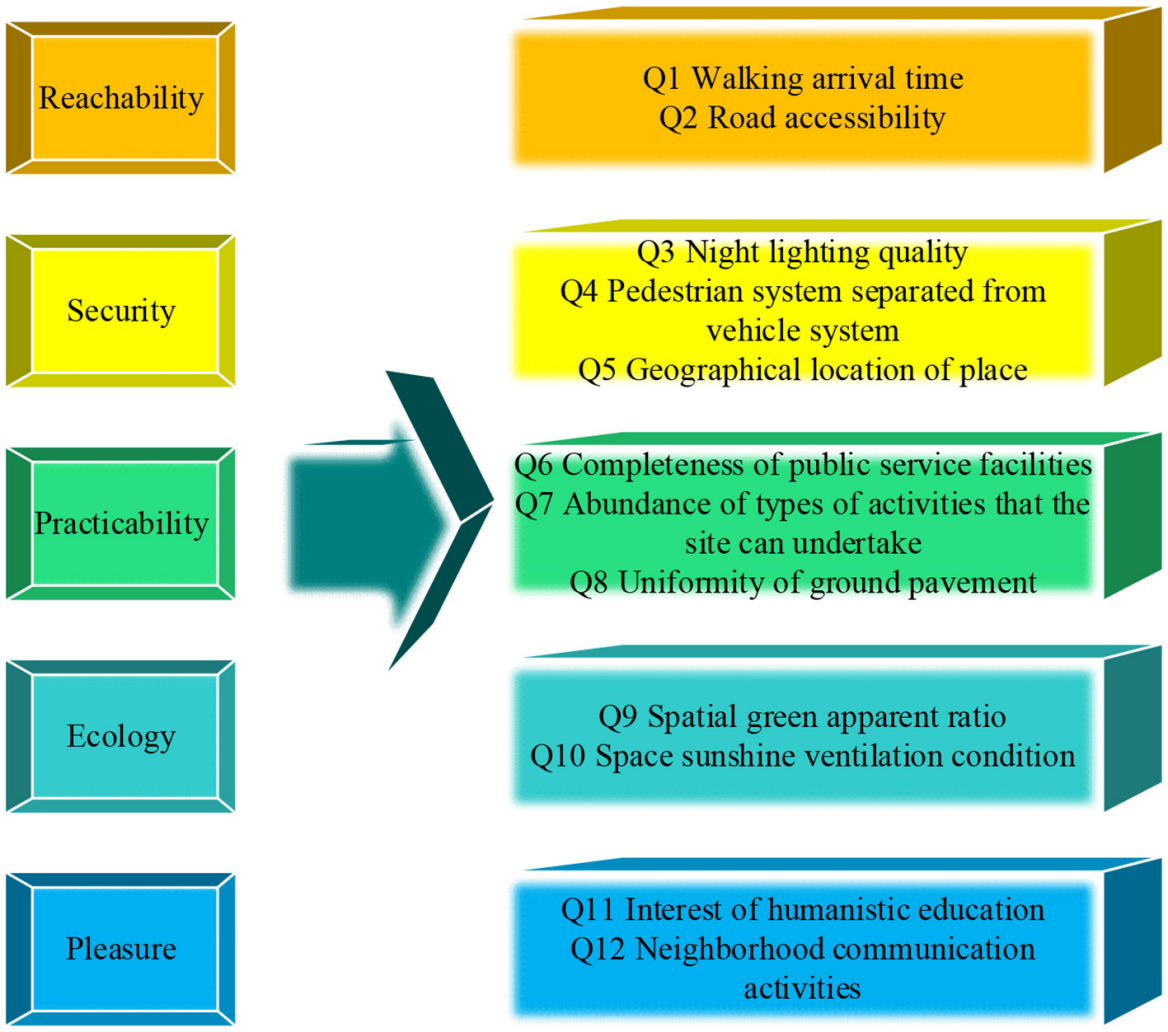
Evaluation model of public space satisfaction in urban settlements.

Under the promotion of ecological economy and LCE, based on PH, the public space of urban settlements should use accessibility, safety, practicality, ecology, and pleasure as the main goals to construct a satisfaction evaluation model of public space in settlements. The details are revealed in Figure 2 (25).

In Figure 2, the satisfaction of public space in urban settlements is evaluated from five aspects: accessibility, safety, practicality, ecology, and pleasure. The specific criteria are: in the comprehensive evaluation of public space in urban settlements, accessibility includes Q1 walking distance time and Q2 road accessibility. Safety mainly includes Q3 night lighting quality, Q4 traffic diversion, and Q5 location. The practicality mainly includes Q6 the completeness of the types of public service facilities, Q7 the richness of the types of activities that the site can undertake, and Q8 the neatness of the ground paving. Ecology mainly includes Q9 space green viewing rate and Q10 space sunshine ventilation conditions. Pleasure mainly includes Q11 humanities education fun and Q12 neighborhood communication activities (26, 27).

MLR models are used for analysis. Suppose there is a linear correlation between the dependent variable *Y* and the *l* independent variable *X*. The model is (28):


(1) $$\begin{array}{l} Y=\gamma_{0}+\gamma_{1}X_{1}+\gamma_{2}X_{2}+\cdots +\gamma_{l}X_{l}+\sigma \end{array}$$

In Eq. (1), γ_0_ is a regression constant. γ_1_, γ_2_, ⋯, γ_*l*_ are regression coefficients. σ is the regression residual.

If *m* observations are made for the independent variable *X* and the dependent variable *Y*, the observations for the *m* group are obtained. Then, its matrix is:


(2) $$\{\begin{array}{c} Y_{1}=\gamma_{0}+\gamma_{1}X_{11}+\gamma_{2}X_{12}+\cdots +\gamma_{l}X_{1l}+\sigma_{1}\\ Y_{2}=\gamma_{0}+\gamma_{1}X_{21}+\gamma_{2}X_{22}+\cdots +\gamma_{l}X_{2l}+\sigma_{2}\\ Y_{m}=\gamma_{0}+\gamma_{1}X_{m1}+\gamma_{2}X_{m2}+\cdots +\gamma_{l}X_{ml}+\sigma_{m} \end{array}$$

In Eq. (2), σ_1_, σ_2_, ⋯, σ_*m*_ is the regression residual.

The questionnaire is conducted here. The reliability of the questionnaire is measured using a reliability factor, which is calculated according to Eq. (3).


(3) $$\begin{array}{l} \alpha =\frac{k}{k-1}\;*\;\left(1-\frac{\sum_{i=1}^{k}S_{i}^{2}}{S_{T}^{2}}\right)\; \end{array}$$

In Eq. (3), *k* is the total number of items in the questionnaire. *S*_*i*_ is the within-question variance of the score for the *i*th question. *S*_*T*_ is the variance of the total score for all questions. The reliability coefficient of the questionnaire should preferably be above 0.8, and between 0.7–0.8 is acceptable.

The Kaiser-Meyer-Olkin (KMO) test is used to verify the validity of the questionnaire. It is calculated according to Eq. (4).


(4) $$\begin{array}{l} KMO=\frac{\sum \sum_{i\neq j}r_{ij}^{2}}{\sum \sum_{i\neq j}r_{ij}^{2}+\sum \sum_{i\neq j}r_{ij^{*}1,2,\cdots ,k}^{2}} \end{array}$$

KMO metrics are as follows. Above 0.9 indicates that it is very suitable; 0.8 indicates that it is suitable; 0.7 means fair; 0.6 indicates that it is not very suitable; below 0.5 indicates that it is extremely unsuitable. The KMO statistic is between zero and one. When the sum of squares of the simple correlation coefficients between all variables is much greater than the sum of squares of the partial correlation coefficients, the KMO value is close to one. The closer the KMO value is to one, the stronger the correlation between the variables and the more suitable the original variable is for factor analysis. When the sum of squares of the simple correlation coefficients between all variables is close to zero, the closer the KMO value is to zero, the weaker the correlation between the variables, and the less suitable the original variables are for factor analysis.

## 4. Experimental design and performance evaluation

### 4.1. Datasets collection

J City is selected as the research object. Settlement A was completed in 1994 with a total of 2,225 households. It is a multi-story building and is a first-class demonstration community for transforming the old city in China. Settlement B was completed in 2005 with a total of 4,129 households. It is a multi-high-rise building and is the first patented settlement in J City. Settlement C was completed in 1998 with a total of 1,943 households. It belongs to multi-story and multi-high-rise mixed buildings. It is also China's first batch of national healthy housing demonstration project communities. A random sample is taken here, and questionnaires are distributed to the selected A, B, and C settlements. Samples are performed for all ages and in clear and rainless weather.

The questionnaire is divided into three main parts. The first part is the basic information of the respondent, such as gender, age, educational background, and length of residence. The second part is the basic situation of outdoor activities, including the frequency of outdoor activities in the community and the time and duration of outdoor activities. The third part is the evaluation of the public space of the settlement, which is carried out from five aspects: accessibility, safety, practicality, ecology, and pleasure. There are 12 second-level indicators from Q1 to Q12. Each second-level indicator has a maximum score of five points, from high to low, five points for very satisfactory, four for more satisfactory, three for general satisfaction, two for less satisfactory, and one for very dissatisfied. Meanwhile, these five aspects will also be used as independent variables of the model. The intensity of outdoor activities of residents in urban settlements is selected as the dependent variable, expressed as the weekly outdoor activity frequency of residents in urban settlements. A total of 300 questionnaires are distributed, 100 per settlement. A total of 284 questionnaires are collected. After removing the blank questionnaires in the retracted questionnaire and the invalid questionnaire with incomplete and regular answers, 269 valid questionnaires were obtained.

### 4.2. Experimental environment and parameters setting

**Figure 3:**
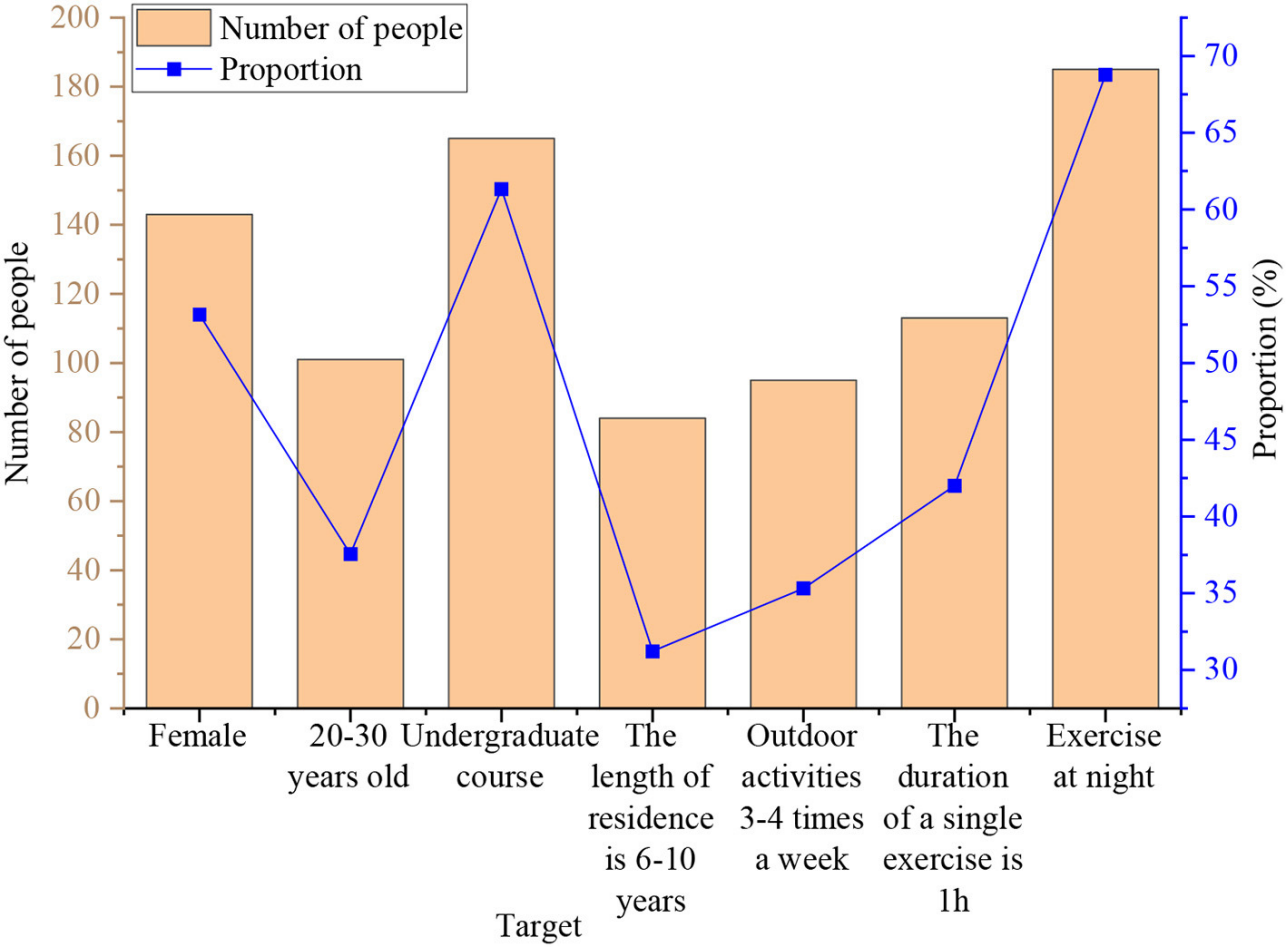
Basic information of interviewed residents.

The basic information of the interviewed residents is demonstrated in Figure 3. There are many projects, so only the one with the most people under each project is selected in the figure.

From Figure 3, of the residents surveyed, 143 are women, accounting for 53.16%. The ratio of men to women is more balanced, with women slightly higher than men. Regarding age, the number of people in the 20–30 age group is the highest. The number of people under 19 and over 61 years old is less, but the age distribution is more widespread overall. Regarding academic qualifications, the number of people with bachelor's degrees is the largest, accounting for 61.34%. In outdoor activities, 35.32% of people are active three–four times a week, 42.01% have a single activity time of 1 h, and 68.77% are used to being active at night.

### 4.3. Performance evaluation

First, the reliability and validity of the questionnaire are tested. Reliability analysis of the data using Statistical Package for the Social Sciences software yields a Cronbach's α coefficient of 0.914 for the comprehensive questionnaire. It is in the high confidence range of 0.7 ≤ α ≤ 0.98. The overall confidence of the data of the explanatory scale is high, which meets the statistical analysis standards. The data is tested for validity after reliability testing. After software calculation, the KMO result is 0.94 > 0.6. In addition, the significance of the Bartley spherical test is *p* = 0.000 < 0.05. It shows that the scale data obtained in this study are suitable for factor analysis and meet the data standards.

**Figure 4:**
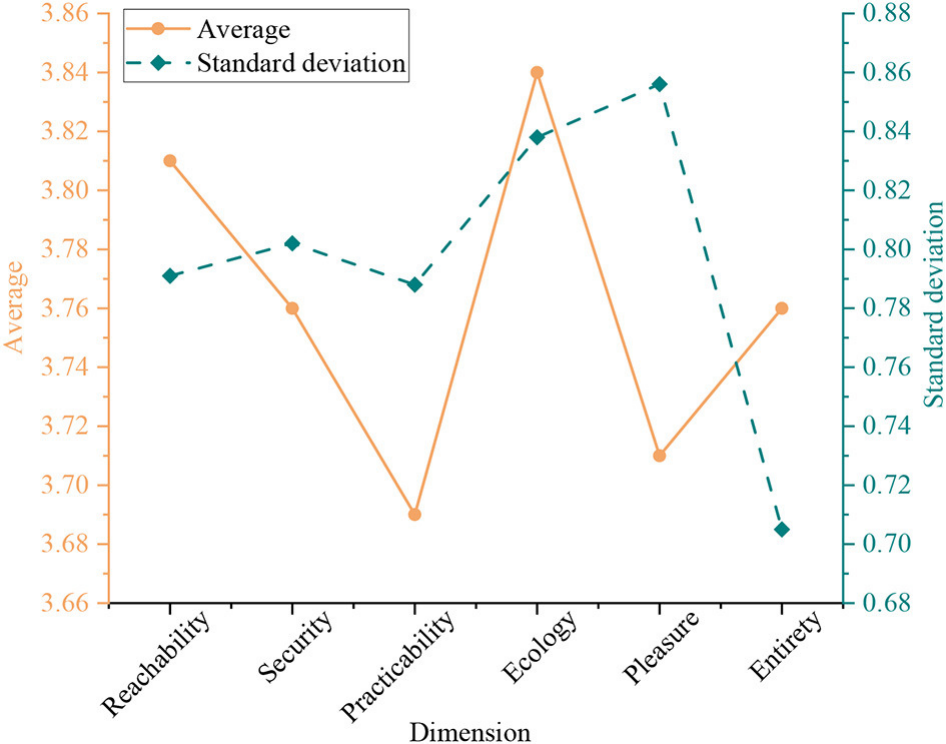
Descriptive statistical analysis results of dependent variables.

Second, descriptive statistics are performed on the questionnaire. The descriptive statistical results for the five dependent variables are shown in Figure 4.

From Figure 4, residents rate accessibility, safety, practicality, ecology, and pleasure with an average score of 3.68. Residents rate ecology and accessibility the most, with average scores of 3.84 and 3.81, respectively. Residents rate practicality the least, with an average of 3.69. Residents' overall rating of these five dimensions is 3.76, with a standard deviation (SD) of 0.705. The SD is 0.705. The mean is high, and the SD is small. It indicates that the residents of the three settlements have a good overall evaluation of the public space in the settlements.

**Figure 5:**
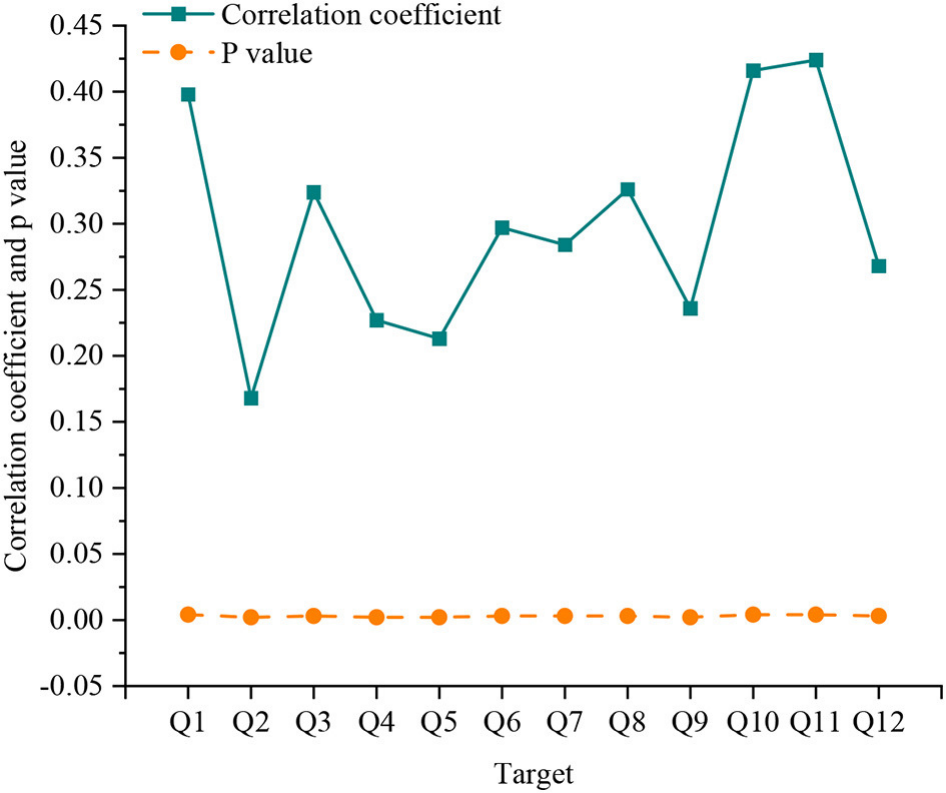
Correlation test between evaluation factors and independent variables of each indicator.

Then, a correlation test between variables is performed. The Pearson correlation coefficient test compares the evaluation factors of Q1–Q12 with the outdoor activity frequency of independent residents. Figure 5 reveals the results.

From Figure 5, the *p*-value of each indicator is < 0.01, so it is sorted by correlation. The correlation coefficients of Q11 humanities education fun, Q10 space sunshine ventilation conditions, and Q1 walking distance time are 0.424, 0.416, and 0.398, respectively. They rank in the top three, indicating that these three indicators have the most significant impact on the frequency of residents' outdoor activities. Overall, the *p*-value of the indicator Q1 to Q12 is < 0.01. The correlation coefficients are all positive. The results show that all 12 independent variables significantly influence the dependent variable, which meets the statistical test criteria.

**Figure 6:**
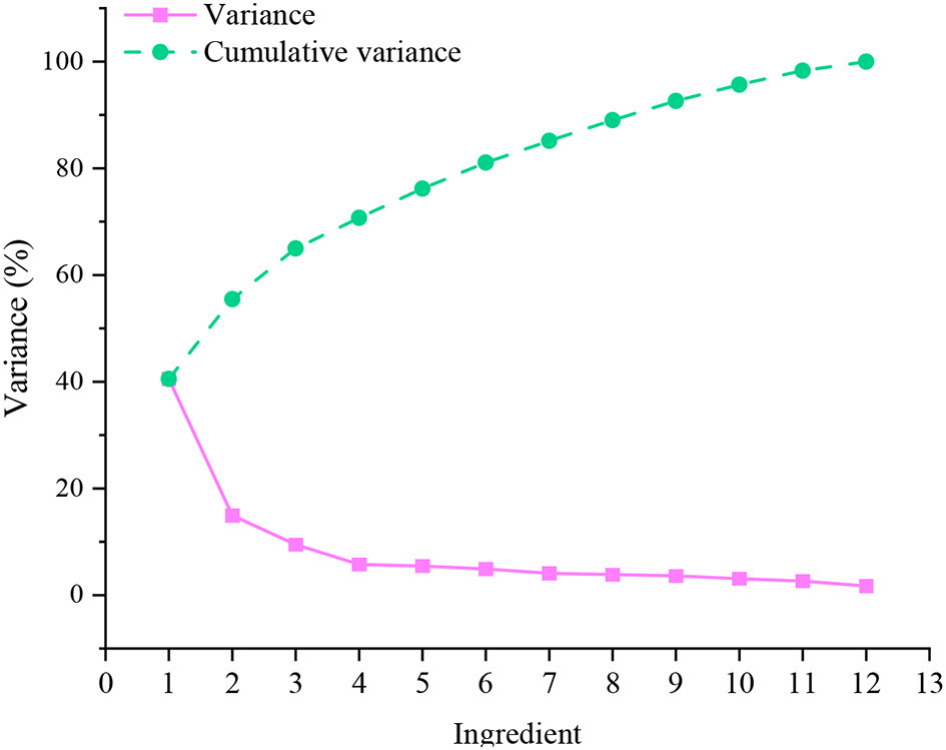
Explanatory total variance statistical results.

Next, MLR analysis is performed on the independent and dependent variables. Explanatory total variance statistics are performed for 12 independent variables. The results are plotted in Figure 6.

**Figure 7:**
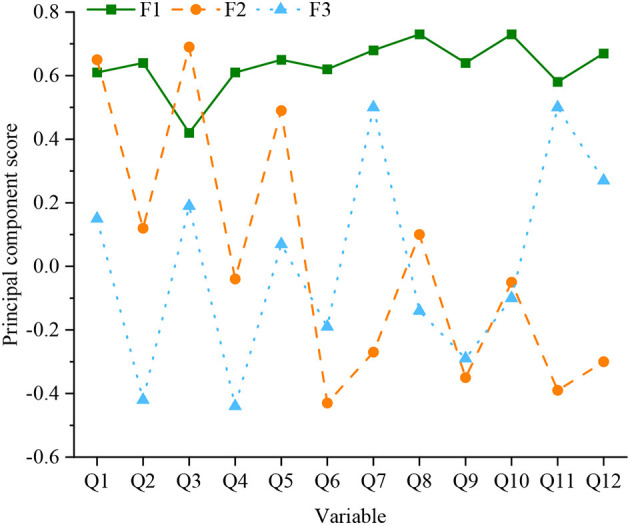
Statistics of principal component score coefficient after rotation.

From Figure 6, among the total explanatory variances obtained by statistics, the explanatory conflicts of the first three principal factors, with values >1 reaching 64.97%. Therefore, it can be determined that the twelve evaluation indicators can be divided into three dimensions, which the three macro principal factors can explain. There is a strong correlation between the original variables in factor analysis. The factor load matrix can be calculated. So, after the maximum orthogonal rotation of its variance, the factor score coefficient matrix and the rotated load matrix can be obtained. The calculation results are shown in Figure 7.

**Figure 8:**
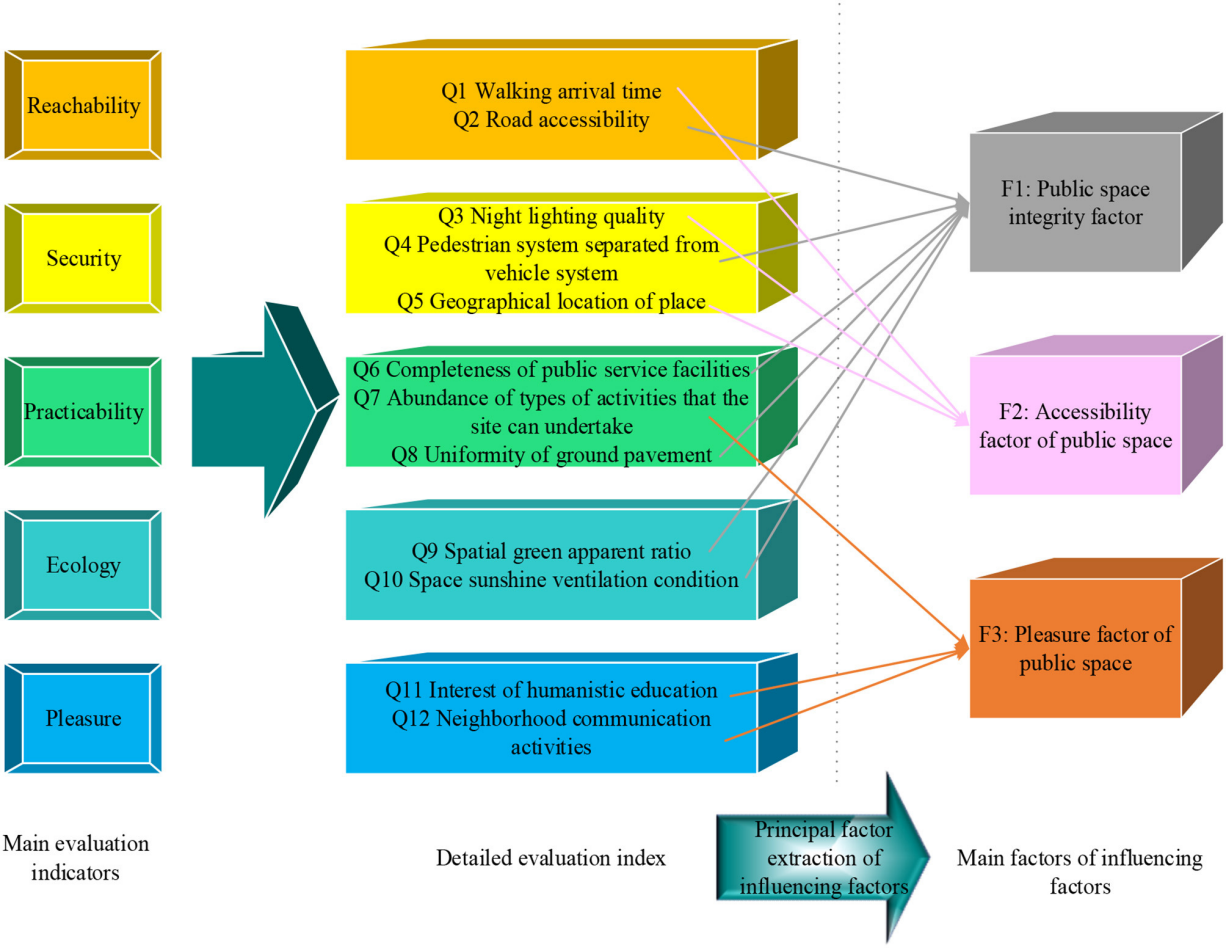
Schematic diagram of the extraction of the principal factor of the influencing factors.

From Figures 6, 7, the principal factor F1 has a large load coefficient in the six original factors of Q2, Q4, Q6, Q8, Q9, and Q10, exceeding 0.6. The variance contribution rate is 24.07%. The principal factor F2 has a large load coefficient in the three original factors of Q1, Q3, and Q5, exceeding 0.75. The variance contribution rate is 44.72%. The principal factor F3 has a large load coefficient in the three original factors of Q7, Q11, and Q12, exceeding 0.69. The variance contribution rate is 64.97%. Through the understanding of the detailed indicators represented by the three principal factors, they are integrated into the renaming work. F1, F2, and F3 are renamed as public space integrity factor, accessibility factor, and pleasure factor, respectively. The cumulative variance contribution rate of the three is 64.97%, which can explain the composition of the influencing factors of the public space of the settlement on the promotion of residents' health activities. The structural model reintegration is displayed in Figure 8.

After extracting the principal factors, MLR analysis is performed on the three macro factors. The MLR equation is *Y* = 0.346*F*_1_ + 0.355*F*_2_ + 0.223*F*_3_ + σ. It can be seen that the accessibility factor F2 of public space has the greatest influence on the outdoor activities of urban residential residents, followed by the integrity factor F1 of public space. The last one is the pleasure factor F3 of public space. Therefore, in the optimal use of public space in settlements, attention should be paid to the integrity and accessibility of public space in urban settlements. Still, the improvement in pleasure should not be ignored.

### 4.4. Discussion

In summary, the factors affecting residents' satisfaction with the public space of the settlement are mainly the accessibility, integrity, and pleasure of the public space. Anastasiou and Manika conducted a field study of residents of a medium-sized Greek city. They surveyed residents on how satisfied they were with urban open space factors. Based on multi-criteria analysis (factor analysis), the factors of residential satisfaction were obtained to capture the shortcomings and dynamic characteristics that shaped urban open space. It was found that residents' satisfaction with urban open space was a function of five factors: the overall operation of open space, the quality of leisure facilities, the contribution of bioclimatic design of large-scale projects, the suitability of infrastructure to children, and respect for local cultural identity (29). Both it and this study confirm that the integrity of public space affects residents' satisfaction with the public space of settlements. Tian et al. chose scenic open spaces in downtown Xi'an, China. The thermal perception (thermal sensation, comfort, and acceptability) of residents and tourists was investigated through meteorological measurements and questionnaires. Physical factors were the main influencing factors of residents' thermal perception, followed by personal, social, and psychological factors (30). Their research angles differ from this paper's, so the results cannot be compared. Jiang and Huang surveyed 7,326 respondents in 78 settlements in Beijing, China. They documented the main characteristics of these residential green spaces and employed multiple logistic regression analyses and multi-level mixed-effects logistic regression analysis. The results showed that having open spaces, gazebos, or shaded paths for numerous activities would significantly increase the likelihood of using residential green area at least once a week (31). The “multi-activity open space, gazebo, or shaded path” is public space accessibility, which is consistent with the findings of this paper. They all show the impact of accessibility in public spaces on satisfaction.

## 5. Conclusion

This paper studies the connotation and essence of the ecological economy and LCE to explore the influencing factors of residents' satisfaction with public space in settlements. Based on the concept of PH, the characteristics of public space in urban settlements are analyzed. Based on five first-level and 12 second-level indicators, the evaluation model of residents' satisfaction with public space in urban settlements is constructed. The following conclusions are obtained through a questionnaire survey and linear regression analysis: (1) Most interviewed residents engage in outdoor activities at night. There will be three–four activities per week lasting 1 h. (2) The humanities education fun, space sunshine ventilation conditions, and walking distance time have the greatest impact on the frequency of residents' outdoor activities. Overall, all 12 independent variables significantly positively affect the dependent variable. (3) Through the principal factor analysis, the principal factors affecting residents' satisfaction with public space in settlements are the accessibility, integrity, and pleasure of public space, and the degree of influence decreases. In summary, the public space resources of urban settlements can be optimized from accessibility, integrity, and pleasure of public spaces in settlements to promote residents to go to public spaces for outdoor activities and physical exercise, which is more conducive to the PH of residents. However, there are some shortcomings. Only three representative settlements of a city are selected, which cannot cover all cities. It remains to be seen whether the research results can be universalized due to the differences between the North and the South. Follow-up studies can select more samples of cities and settlements for further exploration and summary. In addition, in implementing real-world projects, upgrading public spaces in urban settlements cannot be idealized only for health promotion. Practical projects such as the renewal and renovation of old residential areas and the organic renewal of outdoor spaces in residential areas will include transforming public spaces in urban settlements. Follow-up research can start from this aspect to carry out factor correlation research.

## Data availability statement

The raw data supporting the conclusions of this article will be made available by the authors, without undue reservation.

## Ethics statement

The studies involving human participants were reviewed and approved by Hunan City University Ethics Committee. The patients/participants provided their written informed consent to participate in this study. Written informed consent was obtained from the individual(s) for the publication of any potentially identifiable images or data included in this article.

## Author contributions

All authors listed have made a substantial, direct, and intellectual contribution to the work and approved it for publication.
